# The Synthetic Surfactant CHF5633 Restores Lung Function and Lung Architecture in Severe Acute Respiratory Distress Syndrome in Adult Rabbits

**DOI:** 10.1007/s00408-024-00689-z

**Published:** 2024-04-29

**Authors:** Pavol Mikolka, Petra Kosutova, Maros Kolomaznik, Nikolett Nemcova, Juliana Hanusrichterova, Tore Curstedt, Jan Johansson, Andrea Calkovska

**Affiliations:** 1 Department of Physiology, Jessenius Faculty of Medicine in Martin, Comenius University in Bratislava, Martin, Slovakia; 2 Biomedical Centre Martin, Jessenius Faculty of Medicine in Martin, Comenius University in Bratislava, Martin, Slovakia; 3 Department of Molecular Medicine and Surgery, Karolinska Institutet, Stockholm, Sweden; 4 Department of Biosciences and Nutrition, Karolinska Institutet, Neo, Huddinge, Sweden

**Keywords:** Pulmonary surfactant, CHF5633, Surfactant replacement therapy, ARDS model, Lung function, Inflammation

## Abstract

**Purpose:**

Acute respiratory distress syndrome (ARDS) is a major cause of hypoxemic respiratory failure in adults. In ARDS extensive inflammation and leakage of fluid into the alveoli lead to dysregulation of pulmonary surfactant metabolism and function. Altered surfactant synthesis, secretion, and breakdown contribute to the clinical features of decreased lung compliance and alveolar collapse. Lung function in ARDS could potentially be restored with surfactant replacement therapy, and synthetic surfactants with modified peptide analogues may better withstand inactivation in ARDS alveoli than natural surfactants.

**Methods:**

This study aimed to investigate the activity in vitro and the bolus effect (200 mg phospholipids/kg) of synthetic surfactant CHF5633 with analogues of SP‐B and SP‐C, or natural surfactant Poractant alfa (Curosurf^®^, both preparations Chiesi Farmaceutici S.p.A.) in a severe ARDS model (the ratio of partial pressure arterial oxygen and fraction of inspired oxygen, *P/F* ratio ≤ 13.3 kPa) induced by hydrochloric acid instillation followed by injurious ventilation in adult New Zealand rabbits. The animals were ventilated for 4 h after surfactant treatment and the respiratory parameters, histological appearance of lung parenchyma and levels of inflammation, oxidative stress, surfactant dysfunction, and endothelial damage were evaluated.

**Results:**

Both surfactant preparations yielded comparable improvements in lung function parameters, reductions in lung injury score, pro-inflammatory cytokines levels, and lung edema formation compared to untreated controls.

**Conclusions:**

This study indicates that surfactant replacement therapy with CHF5633 improves lung function and lung architecture, and attenuates inflammation in severe ARDS in adult rabbits similarly to Poractant alfa. Clinical trials have so far not yielded conclusive results, but exogenous surfactant may be a valid supportive treatment for patients with ARDS given its anti-inflammatory and lung-protective effects.

## Introduction

Acute respiratory distress syndrome (ARDS) is associated with severe and acute lung inflammation. Aspiration of hydrochloric acid (HCl) may evoke direct damage to the alveolar–capillary membrane and promote polymorphonuclear leukocyte adhesion, activation and sequestration [1]. Mechanical ventilation (MV) as a life-saving intervention is equired in ARDS [2], but improper MV might induce or aggravate lung injury, resulting in ventilator-induced lung injury (VILI). This may lead to an increase in mortality [3–6]. VILI has been linked to the activation of transcription nuclear factor (NF)-κB whose consensus sequence was detected in genes of cytokines and chemokines increased in response to overventilation [7]. Neutrophils, alveolar macrophages, alveolar epithelial cells, and reactive oxygen species have all been shown to be affected in VILI. Activated neutrophils infiltrate the alveoli and release cytotoxic substances and proinflammatory mediators, aggravating inflammation and damaging the alveoli. Tumour necrosis factor-α (TNF-α) and interleukin (IL)-6 are released by macrophages, intensifying the inflammatory reaction. In addition to attracting a significant number of peripheral neutrophils to the lungs and macrophages activation exacerbate the inflammatory lesions present in the lungs [8].

Acute pulmonary damage might disrupt lung surfactant in several ways. Surfactant activity has been impaired in bronchoalveolar lavage fluid (BALF) or tracheal aspirates from patients with ARDS or other conditions involving lung damage [9–13]. Extensive studies have shown that injury-related inhibitors, including plasma and blood proteins [14–19], meconium [20], reactive oxidants [19, 21–23], and lytic enzymes like proteases [24] and phospholipases [25, 26], significantly reduce the activity of lung surfactant. Albumin and other blood proteins mainly decrease surface activity by reducing the number of active surfactant components that may enter the alveolar air–water interface by competitive adsorption [14, 27]. On the other hand, during dynamic compression, fatty acids, lysophospholipids, or lipids found in cell membranes may combine with the surface layer and prevent it from reaching low surface tension (ST) [15, 27, 28]. Surfactant lipids or proteins are chemically altered by phospholipases, proteases, and reactive oxygen and nitrogen species [12, 25, 29]. The well-established fact that surface activity deficits can be mitigated in vitro by increasing the concentration of active surfactant, even in the presence of inhibitor substances, provides a rationale for exogenous surfactant supplementation strategies [30, 31]. Several randomized controlled trials of exogenous surfactant replacement in ARDS have shown no or limited improvement in clinical outcomes [32]. Several factors e.g. poor alveolar distribution, methodology of the study, ARDS heterogeneity, and dysregulated surfactant metabolism remain significant issues in designing clinical trials [33–36].

Randomised controlled experiments have assessed both synthetic and animal-derived surfactants from porcine or bovine sources [37]. When compared to the first or second generations of synthetic surfactants, animal-derived surfactants lead to a quicker weaning from respiratory support, a shorter invasive ventilation period, and a lower death rate [38]. When compared to bovine surfactants, treatment with porcine natural derived Poractant alfa is linked with superior results [39]. This difference in outcome is probably attributable to composition, volume, and/or dosage. CHF5633 (Chiesi Farmaceutici S.p.A.) is the first synthetic surfactant containing both peptide analogues of the two hydrophobic surfactant proteins B (SP-B) and C (SP-C) incorporated into a lipid suspension of phosphatidylcholine and phosphatidylglycerol. Preclinical investigations have demonstrated its resistance to inactivation and physiological effectiveness [40–42]. In the present study, we tested the effects of synthetic surfactant CHF5633 and natural surfactant Poractant alfa on lung function, inflammation and lung architecture in an established experimental model of severe ARDS in adult rabbits. In vitro, the surface activity and function of both surfactant preparations were investigated in the presence of inhibitors in a dynamic system mimicking the respiratory cycle.

## Methods

### Animal Instrumentation

**Fig. 1:**
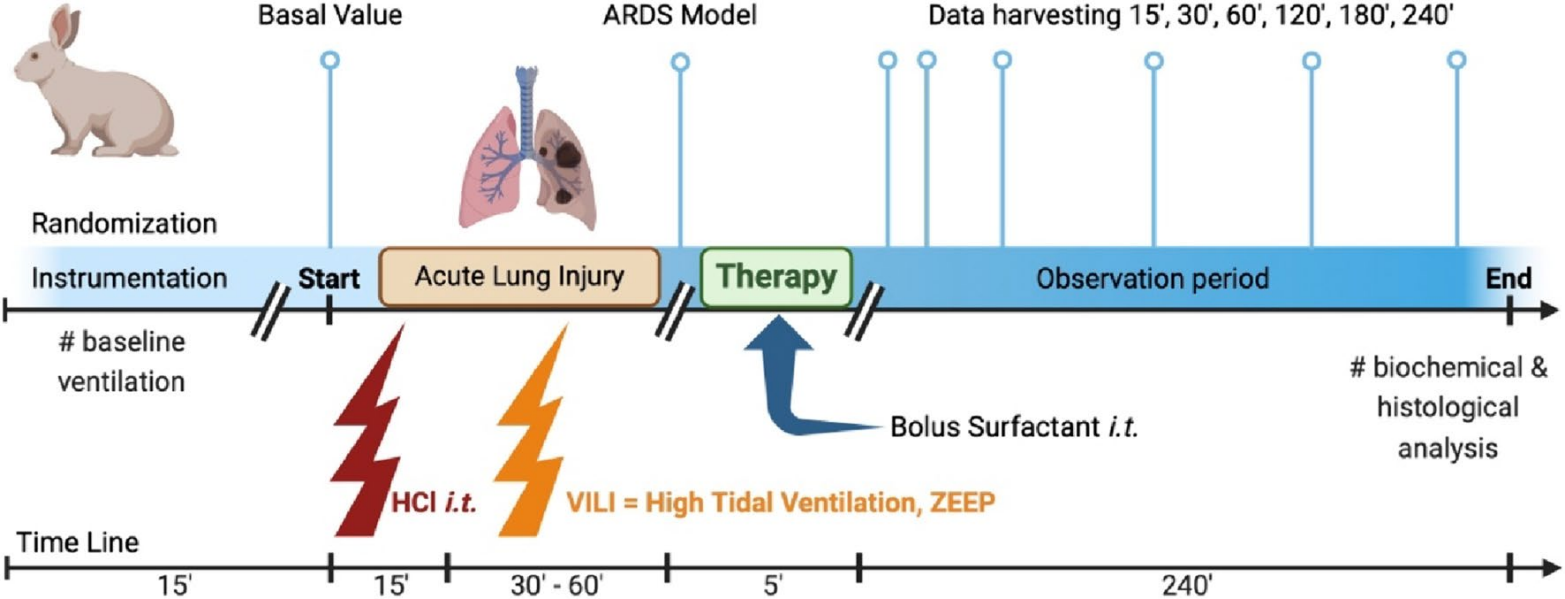
Scheme of the experimental protocol. The procedures were divided into different parts: randomization and instrumentation, induction of lung injury, administration of surfactant, and observation period. Lightning bolts depict single devastating insults to the lungs, such as intratracheal instillation of hydrochloric acid (HCl) and high-tidal ventilation with zero positive end-expiratory pressure (ZEEP) resulting in ventilator-induced lung injury (VILI). The timeline shows the approximate duration of each period in minutes

Both the local Ethics Committee of Jessenius Faculty of Medicine and the National Veterinary Board of Slovakia (EK 6/2021 and Ro. 4590-3/2021-2020, respectively) gave their approval for this study. Twenty-four male adult New Zealand white rabbits aged 15 weeks with 2.5 (SD 0.3) kg body weight (*b.w.*) were used and handled according to the ARRIVE and the Federation of European Laboratory Animal Science Associations (FELASA) guidelines and recommendations, and EU Directive 2010/63/EU for animal experiments. All animals were instrumented in line with previous studies [43, 44]. Figure 1 depicts every step of the experimental protocol.

The animals were first sedated intramuscularly with tiletamine, zolazepam (15 mg/kg *b.w.*; Zoletil, Virbac, France), and xylazine (5 mg/kg *b.w.*; Xylariem, Riemser, Germany) before being placed on a surgical table with a controlled heating temperature of 37 °C in a supine position for the surgical procedures. The right femoral artery and left and right marginal ear veins were cannulated for continuous intravenous (*i.v.*) infusion of anesthetics tiletamine and zolazepam (10 mg/kg *b.w.*/h), Ringer’s acetate solution (10 ml/kg *b.w.*/h), arterial blood sampling, and arterial pressure monitoring. An endotracheal tube was inserted after a tracheotomy was done. After ascertaining adequate anesthesia by the absence of a reaction to aversive stimuli like toe pinching and forceps-pinching abdominal skin, atracurium besylate (0.7 mg/kg *b.w.*/h; Tracrium, Aspen Pharma, Ireland) was used to paralyze animals before they were mechanically ventilated (Aura V, Chirana, Slovakia). Baseline ventilation was delivered in volume-controlled mode with tidal volume (*V*_T_) 6–8 ml/kg *b.w.*, positive end-expiratory pressure (PEEP) of 0.5 kPa, inspiration expiration rate (*I*:*E*) 1:2, respiratory rate (RR) 40 breaths per minute (bpm) and inspiratory oxygen fraction (FiO_2_) of 0.7 for a 20 min stabilization period.

A PowerLab 8/30 multichannel recorder was used to continuously record electrocardiograms with subcutaneous electrodes and invasive arterial pressure monitoring (AD Instruments, Germany). By using a blood gas analyzer (RapidLab TM348, Bayer Diagnostics, Germany), arterial blood samples were used to measure gas exchange and acid–base balance parameters to estimate arterial blood gases (ABG) parameters. Ventilation parameters, e.g. plateau airway pressure (*P*_aw_), static lung-thorax compliance (*C*_stat_), dynamic lung-thorax compliance (*C*_dyn_), mean airway pressure (*P*_aw_), PEEP, and airway resistance (*R*_aw_), were measured by in-build sensors and Aura V ventilator software. The following parameters were calculated: *P*/*F* = a ratio between arterial oxygen partial pressure (PaO_2_) and a fraction of inspired oxygen (FiO_2_); oxygenation index (OI) = (mean airway pressure × FiO_2_)/PaO_2_; and alveolar-arterial gradient (AaG) = [FiO_2_ × (Patm − PH_2_O) − PaCO_2_/0.8] − PaO_2_, where Patm is barometric pressure and PH_2_O is the pressure of water vapour.

### Induction of Experimental Model of ARDS

The combination of acid aspiration and high-tidal volume injurious ventilation resulted in a two-hit experimental model of severe ARDS (Fig. 1). After 20 min of baseline ventilation (*V*_T_ 6 ml/kg *b.w.*, PEEP 0.5 kPa, RR 40 bpm, *I*:*E* 1:2 and FiO_2_ 0.7), respiratory parameters and blood gases were recorded (basal values, BV). FiO_2_ was then increased to 1.0 and the animal received a bolus of hydrochloric acid (HCl, 3 ml/kg *b.w.*, pH 1.25) intratracheally with a 15 min stabilization period as the first insult. Thereafter, in order to simulate ventilator-induced lung injury (VILI), the lungs were ventilated with injurious pattern of high-tidal volumes (HV_T_) with target *V*_T_ 20 ml/kg, zero PEEP, RR 20–30 bpm, *I*:*E* 1:2, and FiO_2_ 1.0. Hypocapnia was tolerated without further RR reduction. During HV_T_ ventilation, ABG were measured every 15 min until the *P*/*F* ratio decreased to 13.3 kPa, which is equivalent to a *P*/*F* of 100 mmHg and classifies our model as intubated severe ARDS according to the new global definition of ARDS [45].

### Surfactant Preparations

The porcine surfactant Poractant alfa (Curosurf^®^) and the synthetic CHF5633 surfactant were used for the treatment. Poractant alfa is an extract of natural porcine lung surfactant. CHF5633 contains *R*-dipalmitoylphosphatidylcholine (DPPC) and 1-palmitoyl-2oleoyl-glycero-3-phospho-1-glycerol (POPG) in a 1∶1 mass ratio 98.3% w/w, and surfactant protein (SP)-B and SP-C analogues (0.2% and 1.5% w/w, respectively). The SP-C analogue is a 33-amino acid protein containing an N-terminal segment analogue of SP-C and a hydrophobic C-terminal helical segment resembling natural SP-C. The SP-C analog’s amino acid sequence is IPSSPVHLKRLKLLLLLLLLILLLILGALLLGL. The SP-B analogue is a 34-amino acid protein derived from two parts (encompassing residues 8–25 and 63–78) of the full-length SP-B [42]. Both surfactant preparations are formulated at a phospholipid concentration of 80 mg/ml and were supplied by Chiesi Farmaceutici S.p.A (Parma, Italy).

### Treatment Protocol

After meeting the criteria for severe ARDS (*P*/*F* < 13.3 kPa), the animals (*n* = 24) were assigned randomly to the following groups: (1) Control group, animals with no surfactant treatment (*n* = 8); (2) Poractant alfa group, animals treated with the natural modified surfactant Poractant alfa (*n* = 8); and (3) CHF5633 group, animals treated with CHF5633 surfactant, respectively (*n* = 8). Surfactant treatment (2.5 ml/kg, 200 mg phospholipids/kg *b.w.*) was given as bolus instillations in the trachea above the carina with the animal in semi‐upright right and left lateral position. In each position, 50% of the dose was administered. In the control group, an air bolus of 2.5 ml/kg was given instead of exogenous surfactant.

After the treatment protocol, the animals were subjected to protective ventilation for additional 4 h in volume‐controlled mode with *V*_T_ 6 ml/kg, PEEP 0.5 kPa, RR 40 bpm, *I*:*E* 1:2, and FiO_2_ 1.0. PEEP was increased gradually up to 1 kPa in cases where oxygen saturation (SaO_2_) fell below 85%. Post-treatment physiological data, including blood gases and respiratory parameters were recorded at 15, 30, 60, 120, 180 and 240 min (Fig. 1). Animals displaying hypotension were given a bolus of saline (10 ml/kg *b.w.*). Finally, 4 h after the treatment, the animals were euthanized under deep anesthesia by receiving an intravenous injection of potassium chloride at a lethal dose. All 24 animals survived the entire protocol.

### Post-mortem Sampling and Analyses

A sternotomy was performed to open the thorax and spontaneously collapsed lungs with clamped trachea above the carina, and the heart were separated from the chest, while *inferior vena cava* and aorta were ligated and transected along with the esophagus. The left lung lobes were lavaged twice with saline (10 ml/kg *b.w.*) to obtain the BALF. Right lung tissue samples were either immediately shock-frozen and stored at −70 °C until biochemical analyses or fixed by immersion in 10% buffered formalin for 2 weeks or used to assess pulmonary edema formation.

In BALF, total and differential white blood cell (WBC) count was estimated using the veterinary hematology analyzer (Sysmex XT-2000i, Sweden). Viability of cells in BALF was determined by automated cell counter Countess™ (Invitrogen, USA) and expressed in percentage.

Then, the BALF was centrifuged (1500 rpm for 15 min) and levels of cytokines, oxidative modification products and other markers were determined in the supernatant. The concentrations of IL-1β, TNFα, IL-6, and IL-8 were quantified using rabbit‐specific ELISA kits (Cloud‐Clone Corp., USA). Protein oxidative damage was determined using the OxiSelectTM Nitrotyrosine ELISA Kit and Advanced Oxidation Protein Products (AOPP) Assay and the OxiSelect™ TBARS Assay Kit was used to detect oxidation of lipids expressed as the concentration of thiobarbituric acid reacting substances (TBARS) (all kits were purchased from Cell Biolabs, USA). Soluble receptor for advanced glycation end products (sRAGE) (MyBioSource, USA), activity of secretory phospholipase A2 (sPLA), and myeloperoxidase activity (MPO) (both Abcam plc., UK) were quantified using rabbit-specific assays. All analyses were performed in duplicate according to the manufacturer’s instructions.

Tissue samples from the right lung from apical, medial, and caudal regions were collected according to a pre-set scheme for estimation of the wet-to-dry (*W*/*D*) lung weight ratio, the extent of lung edema. Lung strips were weighed before and after drying in an oven at 50 °C for 1 week to calculate the *W*/*D* ratio. Total protein content in BALF was determined in supernatant by the Bradford colorimetric method.

Formalin‐fixed lung samples from caudal medial right lung were embedded in paraffin, sectioned, and stained with hematoxylin and eosin. Histological analysis was performed blindly by a veterinary pathologist (SM) and scored according to: neutrophil infiltration, interstitial congestion and hyaline membrane represented 1—normal lung, 2—moderate, 3—intermediate, 4—severe; perivascular edema, emphysema 0—absent, 1—mild-moderate, 2—moderate-severe, 3—severe; for haemorrhage, atelectasis 0—absent, 1—present. The sum of scores was used to assess the total lung injury score as described previously [46].

### Surface Activity Analysis

Natural surfactant was isolated from BALF by taking the supernatant from the first centrifugation (5 min at 1500 × *g* to removed cells) and subjecting it to a centrifugation of 40,000 × *g* at 4 °C (for 65 min to generate a surfactant pellet). The pellet from this high-speed centrifugation was resuspended in 500 µl of saline and determined the lipid content (Lipid Quantification Kit, STA-617, Cell Biolabs, Inc., San Diego, USA). After resuspension of the surfactant pellet, the biophysical activity of the surfactant material from animal model was assessed at a final total lipid concentration of 3 mg/ml. In contrast, exogenous surfactant sample contained 2.5 mg phospholipids/ml.

A pulsating bubble surfactometer (PBS; General Transco Inc., Seminole, FL, USA) was used to assess the surface activity of surfactant preparations. A diluted exogenous surfactant sample was filled in an acrylic sample chamber, preheated to 37 °C. A bubble with a 0.4 mm minimum radius was created and maintained for 30 s of stabilization. Subsequently, pulsation between bubble radius 0.4 and 0.55 mm (corresponding to 50% area expansion and compression) was initiated at a cycling frequency of 20 rpm. Pressure across the bubble’s surface was continuously recorded using a microprocessor. Surface tension values at the minimum and maximum bubble sizes were calculated using the Laplace equation [47]. Plasma proteins (albumin, fibrinogen) were used to evaluate the resistance of surfactants to inactivation in PBS.

### Statistics

Statistical analysis was performed using the statistical software Prism 9 (GraphPad, USA). The results are presented as mean and standard deviation (SD). Data normality was tested using the Shapiro–Wilk test. All measured variables, except semiquantitative histopathological features, had a normal distribution within each group; therefore, two-way ANOVA with Tukey’s post-hoc test in parameters with dynamic changes for specific time-points and Kruskal–Wallis non-parametric test (few variables per group) for testing the differences between the groups were performed. Semiquantitative data from histological evaluation were tested by Chi-square test or Fisher’s exact test for qualitative binary variables (absent/present) and Mann–Whitney non-parametric test for multiple variables (1–4 scale). A *p* value below < 0.05 was considered as statistically significant. The confidence interval (CI) was additional information about the likely magnitude of the effect being investigated and the reliability of the estimate.

## Results

### Pulsating Bubble Surfactometer Analysis

**Fig. 2:**
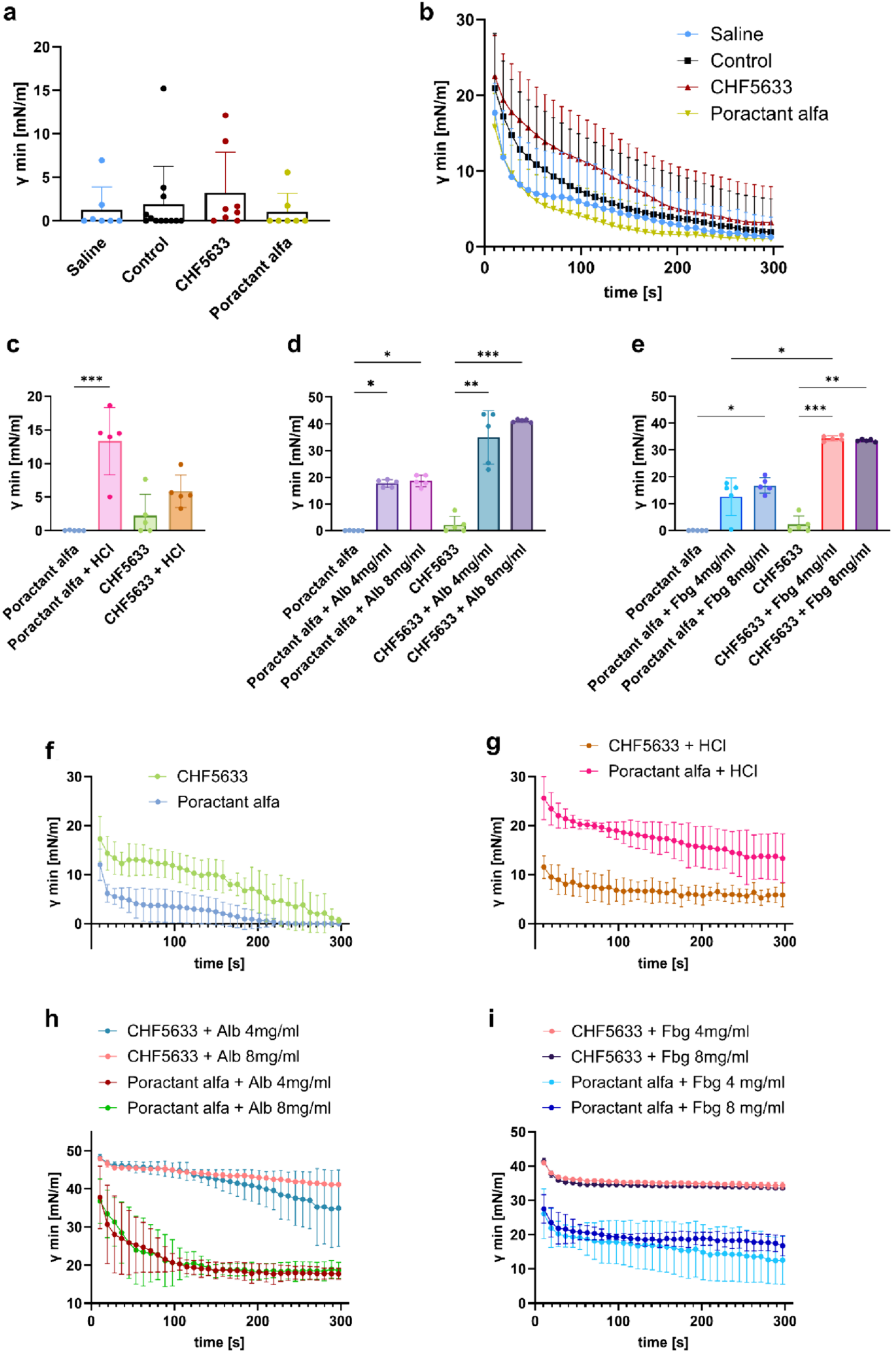
Surface activity and analysis of the surfactant resistance to inactivation. Minimum surface tension (ST; γmin) of **a** surfactant materials isolated from rabbit lavage fluids, **c** minimum ST of the Poractant alfa and CHF5633 surfactant at concentration 2.5 mg/ml mixed with hydrochloric acid (HCl; pH 1.25), and **d** albumin (Alb) and **e** fibrinogen (Fbg) both at concentration 4 or 8 mg/ml after 5 min of pulsation in a pulsating bubble surfactometer (PBS). Minimum ST during the whole analysed period for 5 min of cycling in PBS of **b** surfactant materials from rabbits, **f** the Poractant alfa and CHF5633 at concentration 2.5 mg/ml, **g** Poractant alfa and CHF5633 mixed with HCl (2.5 mg PL/ml, pH 1.25), and **h** mixture of surfactants and albumin (Alb), **i** fibrinogen (Fbg) at concentration 4 mg/ml or 8 mg/ml. Data are presented as mean and SD. Statistical comparisons: *p < 0.05, **p < 0.01, ***p < 0.001

The biophysical activity of the surfactant material recovered from the lungs of the rabbits with ARDS evaluated in the pulsating bubble surfactometer (PBS) showed a tendency to lower the surface tension (ST), which failed to reach statistical significance (Control vs. Saline *p* = 0.964). Significant differences were not observed between treated groups compared to Control (Fig. 2a). The dynamic changes of minimal ST of surfactant material obtained from rabbit lung lavage fluids across the whole period (5 min) of cycling in PBS are shown in Fig. 2b. Poractant alfa and CHF5633 surfactant mixed with HCl (2.5 mg PL/ml, pH 1.25) have a significant effect on the minimum ST only for Poractant alfa (Fig. 2c). Addition of albumin or fibrinogen at both concentrations 4 mg/ml and 8 mg/ml significantly increased min ST of the Poractant alfa and CHF5633 after 5 min of pulsation (Fig. 2d, e). We observed significant difference between Poractant alfa with fibrinogen compared to CHF5633 surfactant with fibrinogen both in 4 mg/ml (Fig. 2e). The dynamic changes of minimum ST of the Poractant alfa and CHF5633 surfactant alone or mixed with HCl, albumin and fibrinogen during the whole period of cycling in PBS are shown in Fig. 2f–i.

### Lung Function Parameters

**Fig. 3:**
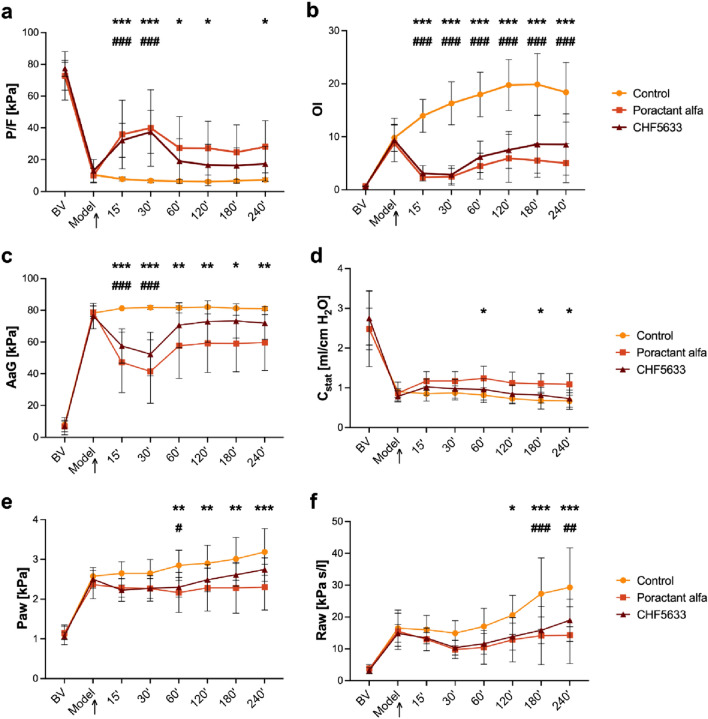
Lung function parameters. **a** The ratio of arterial oxygen partial pressure to fraction of inspired oxygen (*P*/*F*, kPa), **b** oxygenation index (OI), **c** alveolar–arterial gradient (AaG, kPa), **d** static compliance (*C*_stat_, ml/cm H_2_O), **e** mean airway pressure (*P*_aw_, kPa), **f** airway resistance (*R*_aw_, kPa s/l) before (basal value, BV), at established ARDS condition (Model), and during 4 h after administration of surfactant therapy (marked with an arrowhead) in the control, Poractant alfa, CHF5633 groups (*n* = 8 in each group). Data are presented as mean and SD. Statistical comparisons: for Poractant alfa *p < 0.05, **p < 0.01, ***p < 0.001 vs. Control and for CHF5633 ^#^*p* < 0.05, ^##^*p* < 0.01, ^###^*p* < 0.001 vs. Control

The entry lung function parameters in the initial phase of the setup did not differ among the experimental groups (Control vs. Poractant alfa vs. CHF5633) at baseline values (BV) as well as at Model time point (*p* > 0.05 for each parameter). A model of ARDS was established by instillation of hydrochloric acid followed by injurious high-volume ventilation and no PEEP. Induction of lung injury was accompanied by severe deterioration in all measured lung function parameters, including *P*/*F*, OI, AaG, *C*_stat_, *P*_aw_ and *R*_aw_; significant deterioration (*p* < 0.001 for each parameter) was observed at time point Model (representing severe ARDS with *P*/*F* < 13.3 kPa) compared to BV. Deterioration of all lung function parameters persisted only in the untreated control group till the end of the experiment, for additional 4 h (Fig. 3).

Treatment with either surfactant preparation improved lung function, with significant effect at least immediately after administration. Noticeable improvement in *P*/*F*, OI and AaG was observed at 15 and 30 min after therapies with either Poractant alfa or CHF5633 (for all parameters at each time point, *p* < 0.001); however only with Poractant alfa this significant effect persisted till the end of experiment (Fig. 3a–c). In *C*_stat_, *P*_aw_ and *R*_aw_, the effect of treatment was evident and slightly delayed (from 60 min after administration of surfactant), especially for Poractant alfa. Poractant alfa, but not CH5633 surfactant, significantly improved *C*_stat_ (*p* < 0.05) relative to the controls for about the entire observation period (Fig. 3d). CHF5633 improved *P*_aw_ only at 60 min (*p* < 0.05) and *R*_aw_ at 180 min (*p* < 0.001) and 240 min (*p* < 0.01) after administration compared to controls (Fig. 3e, f).

No statistically significant differences were observed between the two surfactant therapies. However, when area-under-the-curve (AUC) analysis was used, AUC of Poractant alfa in *C*_stat_ showed significant differences compared to the CHF5633 (*p* = 0.022, CI: −2.36, −0.11). In addition, only AUC of Poractant alfa differed significantly from controls for *P*/*F* (*p* = 0.031, CI: −118.3, −5.47), AaG (*p* = 0.01, CI: 18.43, 131.9), *C*_stat_ (*p* = 0.003, CI: −2.47, −0.54) and *P*_aw_ (*p* = 0.016, CI: 0.46, 4.53). AUC of CHF5633 therapy in OI (*p* < 0.001, CI: 25.97, 61.42) and *R*_aw_ (*p* = 0.047, CI: 0.44, 58.62) differed from the controls and the effect was comparable with Poractant alfa.

### Arterial Blood Gases (ABG) Parameters

**Fig. 4:**
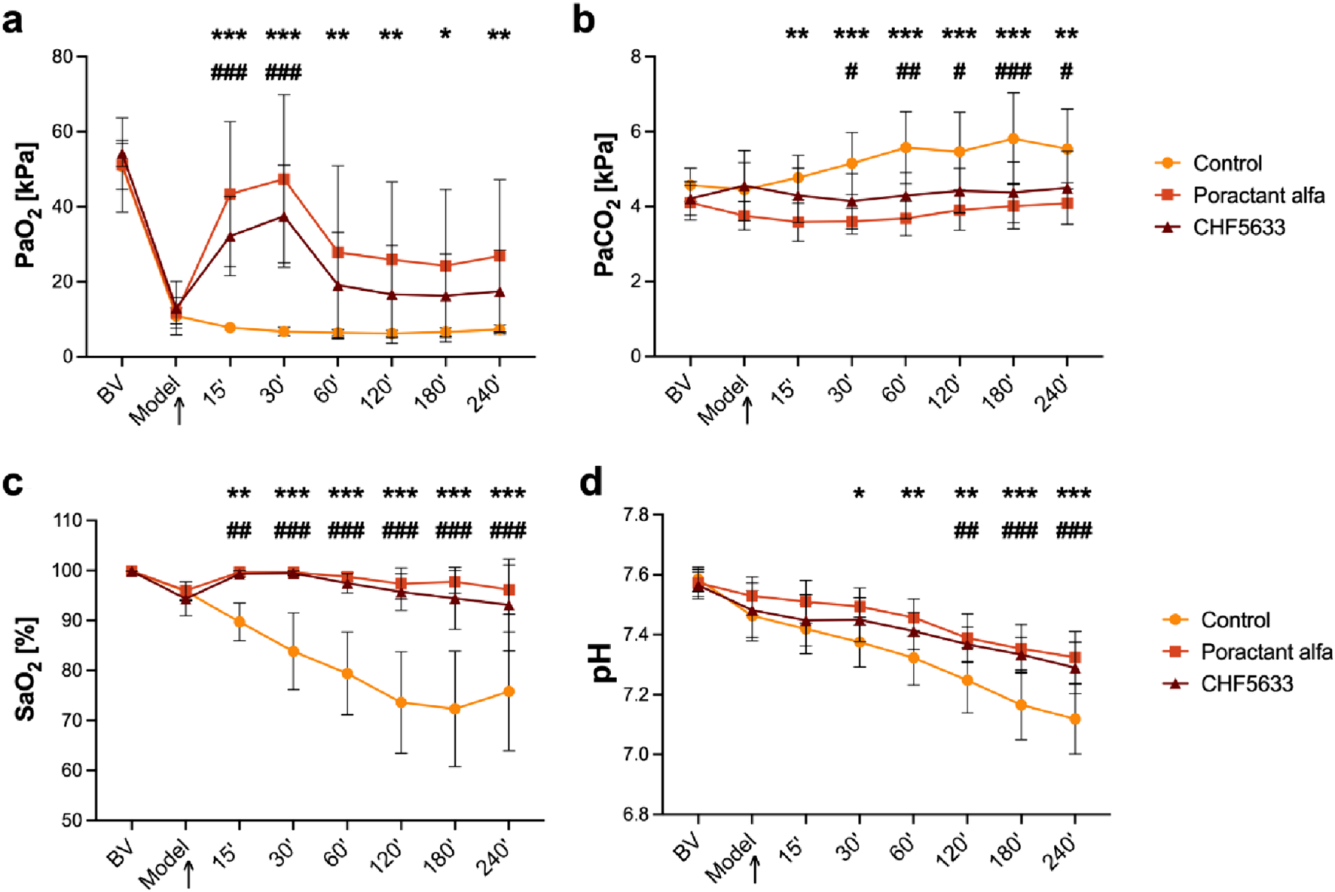
Arterial blood gases (ABG) parameters. **a** Partial pressure of oxygen (PaO_2_, kPa), **b** partial pressure of carbon dioxide (PaCO_2_, kPa), **c** oxygen saturation (SaO_2_, %), **d** pH before (basal value, BV), at established ARDS condition (Model), and during 4 h after administration of surfactant therapy (marked with an arrowhead) in the Control, Poractant alfa, CHF5633 groups (*n* = 8 in each group). Data are presented as mean and SD. Statistical comparisons: for Poractant alfa *p < 0.05, **p < 0.01, ***p < 0.001 and for CHF5633 ^#^*p* < 0.05, ^##^*p* < 0.01, ^###^*p* < 0.001 vs. Control

Administration of either surfactant preparation significantly improved ABG parameters. Poractant alfa significantly improved PaO_2_, PaCO_2_, SaO_2_ and pH immediately after therapy with persisted effect till the end of the 4 h observation period compared to controls (*p* < 0.001 repeatedly for all observed time-points) (Fig. 4). For CHF5633, marked and significant improvement was observed in PaO_2_ only 15 and 30 min after administration compared to controls (for both time-points *p* < 0.001). Compared to controls, CHF5633 improved SaO_2_ throughout the experiment (at each time point *p* < 0.001), PaCO_2_ (markedly at 180 min *p* < 0.001) and pH with delay effect from 120 min after therapy till the end (*p* < 0.01). Poractant alfa had more persistent effects compared to the untreated control animals than CHF5633, but there were no differences between the surfactant treated groups.

### Total and Differential Leukocyte Counts

**Fig. 5:**
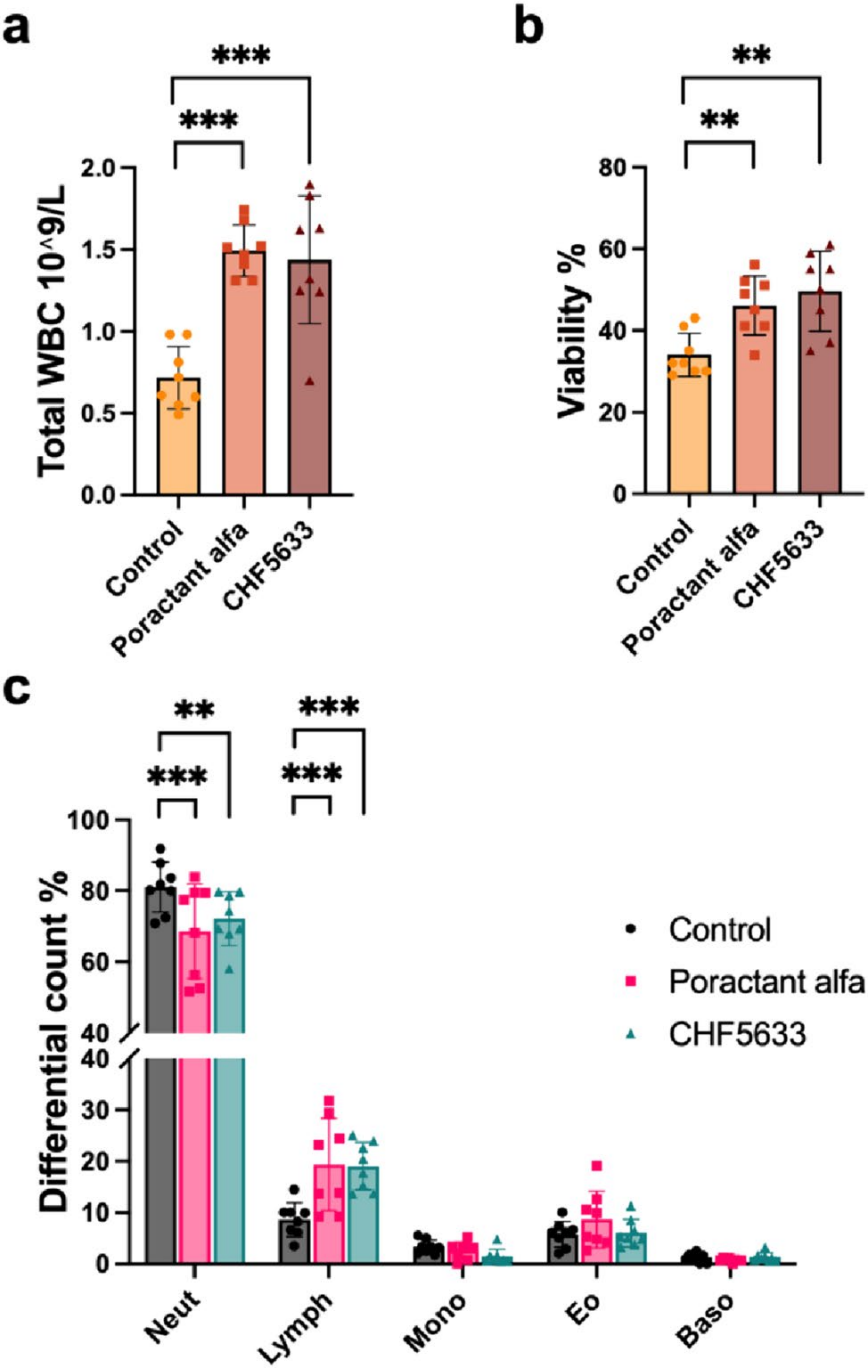
Leukocyte counts. Total (**a**) and differential (**c**) white blood cells (WBC) counts and **b** percentage of viable cells in the bronchoalveolar lavage fluid (BALF) in the control group, and groups treated with Poractant alfa and CHF5633 surfactant (*n* = 8 in each group). *Neut* neutrophils, *Lymph* lymphocytes, *Mono* monocytes, *Eo* eosinophils, *Baso* basophils. Data are presented as individual values with mean and SD. Statistical comparisons: for Poractant alfa and CHF5633 vs. control **p < 0.01, ***p < 0.001

Both surfactant preparations affected total and differential WBC count in the BALF. Significantly increased total WBC count in BALF was found after Poractant alfa (*p* < 0.001, CI: −1.09, −0.46) and CHF5633 (*p* < 0.001, CI: −1.04, −0.40) compared to controls (Fig. 5a). Also, the percentage of neutrophils and lymphocytes changed after both surfactant therapies. Decreased neutrophils after the Poractant alfa treatment (*p* < 0.001, CI: 5.92, 18.93) and CHF5633 (*p* = 0.004, CI: 2.39, 15.41); and increased lymphocytes after Poractant alfa (*p* = 0.001, CI: −17.23, −4.22) and CHF5633 (*p* = 0.001, CI: −16.95, −3.93) compared to controls were observed (Fig. 5c). Cell viability in BALF was significantly higher after administration of either surfactant preparation compared to the untreated controls, for Poractant alfa (*p* = 0.009, CI: −21.20, −3.05), for CHF5633 (*p* = 0.001, CI: −24.70, −6.55) (Fig. 5b). There were no differences in the counts of WBC between the two surfactant preparations.

### Markers of Inflammatory, Oxidative, and Vascular Modifications and Degrading Enzymes

**Fig. 6:**
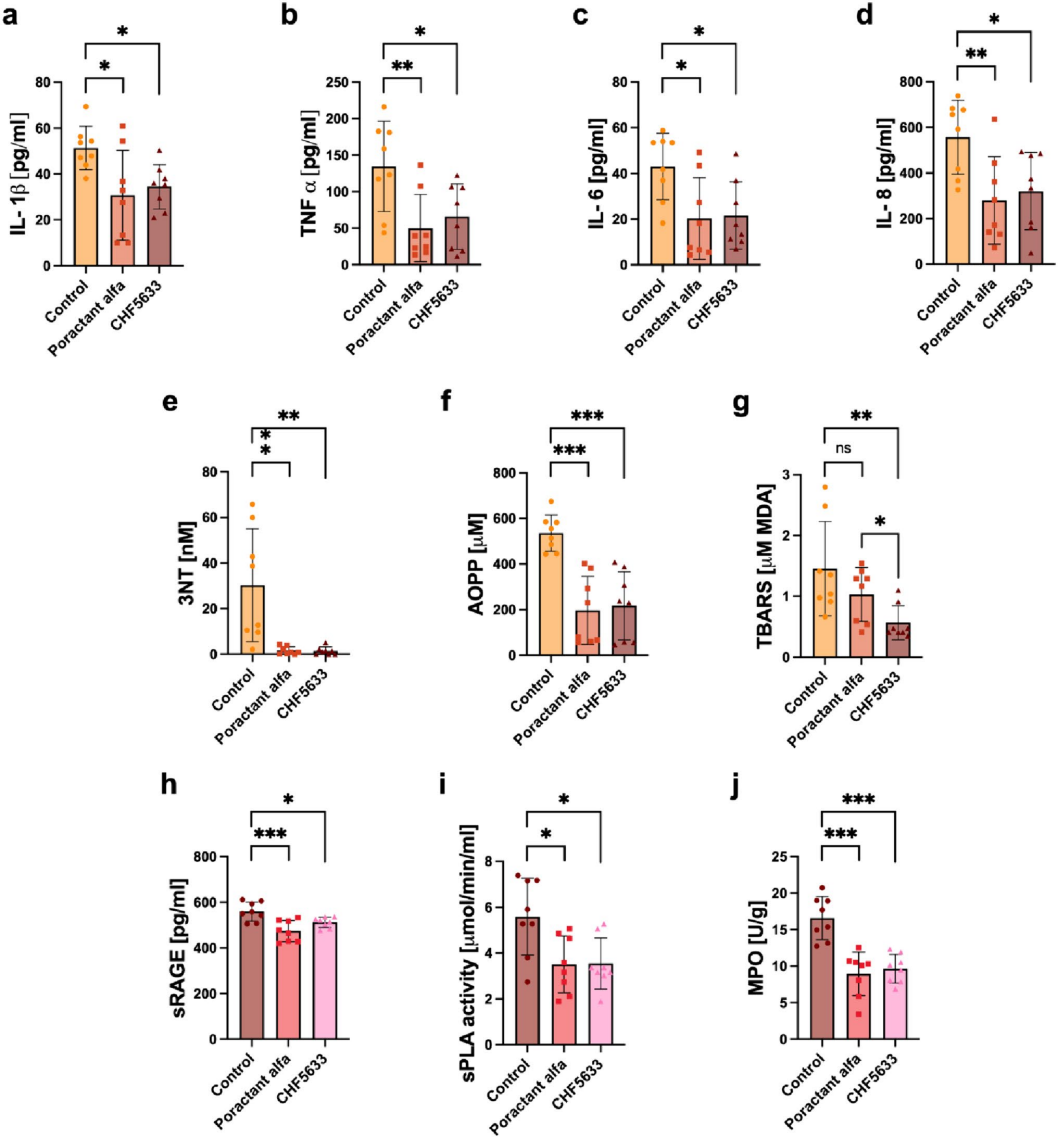
Inflammatory, oxidative, and vascular damage markers and degrading enzymes. Levels of interleukins **a** IL-1β, **b** TNFα, **c** IL-6, **d** IL-8 (all in pg/ml), and **e** 3-nitrotyrosine (3NT, in nM), **f** advance oxidation protein products (AOPP in µM), **g** thiobarbituric acid-reactive substances (TBARS, in µM MDA), and **h** soluble receptor for advanced glycation end products (sRAGE in pg/ml), **i** activity of secretory phospholipase A2 (sPLA in µmol/min/ml), **j** myeloperoxidase activity (MPO, U/g) in BALF of the Control, Poractant alfa, CHF5633 groups (*n* = 8 in each group). Data are presented as individual values with mean and SD. Statistical comparisons: for Poractant alfa vs. CHF5633 vs. control *p < 0.05, **p < 0.01, ***p < 0.001

Both surfactant therapies resulted in reduced levels of proinflammatory cytokines and markers of oxidative modification compared to the untreated control group in BALF. Poractant alfa and CHF5633 significantly decreased levels of cytokines. No marked differences were observed between the two surfactant preparations, even though Poractant alfa appeared to be more efficient; *p*-values for Poractant alfa: IL-1β (*p* = 0.013, CI: 4.34, 36.91), TNFα (*p* = 0.007, CI: 22.5, 146.5), IL-6 (*p* = 0.017, CI: 3.98, 41.47), IL-8 (*p* = 0.009, CI: 66.71, 487.5); and *p*-values for CHF5633: IL-1β (*p* = 0.042, CI: 0.59, 33.16), TNFα (*p* = 0.028, CI: 6.78, 130.8), IL-6 (*p* = 0.025, CI: 2.65, 40.13), IL-8 (*p* = 0.026, CI: 26.18, 447.0) compared to untreated controls (Fig. 6).

Oxidative damage to proteins expressed by 3NT and AOPP was similarly and significantly reduced after administration of both surfactant preparations, for both preparation and both markers *p* < 0.01 compared to untreated controls. Only CHF5633 markedly reduced oxidative damage to lipids expressed by TBARS compared to controls (*p* = 0.007, CI: 0.25, 1.53), in addition, compared to Poractant alfa (*p* = 0.025, CI: −0.86, −0.07) (Fig. 6g).

The level of sRAGE and activity of the enzymes sPLA and MPO were mitigated after both surfactant treatments. Compared to Control group, for Poractant alfa sRAGE (*p* = 0.0004, CI: 40.29, 130.6), sPLA (*p* = 0.012, CI: 0.44, 3.73), MPO (*p* < 0.0001, CI: 4.47, 10.79), and for CHF5633 sRAGE (*p* = 0.041, CI: 1.74, 92.02), sPLA (*p* = 0.014, CI: 0.40, 3.69), MPO (*p* < 0.0001, CI: 3.75, 10.08). No statistically significant differences in biochemical markers except TBARS were observed between the two surfactant therapies (Fig. 6).

### Lung Edema and Protein Content in BALF

**Fig. 7:**
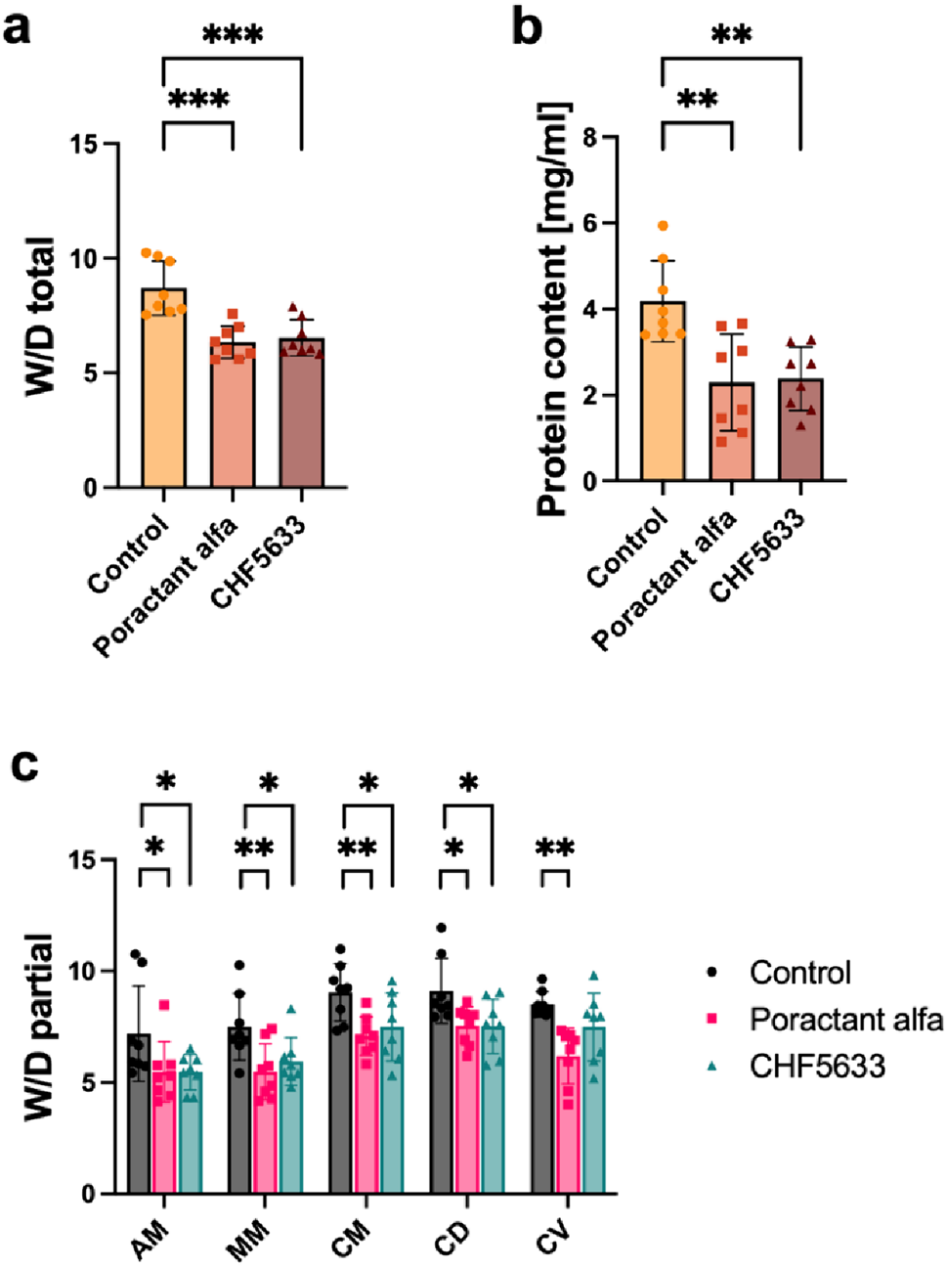
Effects on ARDS-induced deterioration of alveolar-capillary membranes. Total **a** and partial lung edema formation **c** expressed as wet–dry (*W*/*D*) lung weight ratio and **b** protein content in BALF (mg/ml) in the Control group, and groups treated with Poractant alfa or CHF5633 surfactant (*n* = 8 in each group). *AM* apical medial, *MM* medial medial, *CM* caudal medial, *CD* caudal dorsal, *CV* caudal ventral. Data are presented as individual values with mean and SD. Statistical comparisons: for Poractant alfa vs. CHF5633 vs. control *p < 0.05, **p < 0.01, ***p < 0.001

Recovery of the BALF was without difference in all groups (Poractant alfa vs. CHF5633 vs. control, *p* > 0.05). Total protein content in BALF increased in the untreated control group, and, similarly, lung edema expressed as a wet–dry lung weight ratio (*W*/*D* ratio) was increased. Both surfactant preparations resulted in significantly reduced levels of BALF protein content (for Poractant alfa *p* = 0.0013, CI: 0.76, 3.01; for CHF5633 surfactant *p* = 0.002, CI: 0.68, 2.92). Both surfactant therapies decreased the total *W*/*D* ratio compared to controls (for Poractant alfa *p* < 0.0001, CI: 1.27, 3.43; for CHF5633 surfactant *p* = 0.0002, CI: 1.08, 3.24) (Fig. 7a, b). In each section of the lung, both surfactant preparations significantly decreased *W*/*D* with a stronger effect for the Poractant alfa; the most lung parts *p* < 0.01 including caudal ventral part compared to Control group (for CHF5633 *p* < 0.05) (Fig. 7c).

### Histological Evaluation of Lung Tissue

**Fig. 8:**
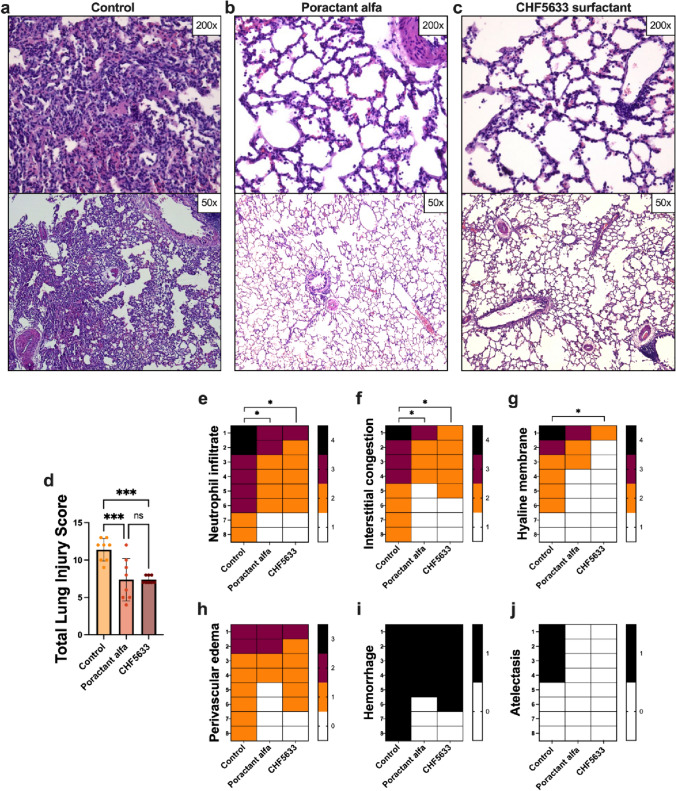
Histological analysis and histopathological quantitation. Lung sections from animals in untreated Control group (**a**), group treated with Poractant alfa (**b**) or CHF5633 surfactant (**c**) (*n* = 8 in each group); the quantification of total lung injury (**d**), neutrophil infiltrate (**e**), interstitial congestion (**f**), hyaline membrane (**g**), perivascular edema (**h**), hemorrhage (**i**), atelectasis (**j**). Heatmap representation of the results of blinded semiquantitative analysis and the color of each cell representing the semiquantitative scoring of the histopathological features of one animal according to scoring systems. In the Control group, the pulmonary parenchyma displayed a diffuse inflammatory cell infiltrate (polymorphs, neutrophils and plasma cells with activated pneumocytes, and erythrocytes) and collapsed alveoli. Massive hyaline membranes and protein debris were observed (**a**). In Poractant alfa group, the lung seemed normal at low power, but slightly thickened alveolar septa displayed rare inflammatory cell infiltrate, mostly polymorphs—neutrophils, plasma cells, and few erythrocytes. The alveolar spaces were airy with inconspicuous protein debris (**b**). In CHF5633 surfactant group, lung parenchyma showed normal alveoli with slightly thickened alveolar septa and thin chronic inflammatory infiltrate, scattered polymorphs, plasma cells and erythrocytes. Inconspicuous hyaline membranes and deposits of proteinaceous material in the alveolar septa were present (**c**). The magnification ×50 and ×200 was used. Statistical comparisons: for Poractant alfa vs. CHF5633 vs. control *p < 0.05, **p < 0.01

In lung tissue, histological signs of severe acute lung injury were observed in the untreated Control group and the alveoli and the pulmonary parenchyma displayed a diffuse miscellaneous inflammatory cell infiltrate. Typical partially disrupted tissue architecture with collapsed alveoli was present. Alveoli displayed an acute cell reaction of polymorphs, predominantly neutrophils and plasma cells with activated pneumocytes, and numerous erythrocytes. In some places, massive hyaline membranes and protein debris were present at the alveolar surface (Fig. 8a). Both surfactants improved the lung architecture. In Poractant alfa group, the lung seemed normal at low power, but slightly thickened alveolar septa displayed rare inflammatory cell infiltrates. There were mostly polymorphs—neutrophils, plasma cells and a few erythrocytes. The alveolar spaces were airy with inconspicuous protein debris. The peribronchial space around the terminal bronchi displayed increased lymphocyte aggregates (Fig. 8b). Similarly, in CHF5633 group, the pulmonary parenchyma showed normal alveoli with slightly thickened alveolar septa and thin inflammatory infiltrates. The alveoli were airy with scattered polymorphs, plasma cells and erythrocytes. Inconspicuous hyaline membranes and deposits of proteinaceous material in the alveolar septa were present (Fig. 8c).

Morphological analysis revealed marked deterioration in all histopathological features in Control group, measured by a scoring system. Both surfactant preparations significantly attenuated neutrophil infiltration (for Poractant alfa *p* = 0.044, for CHF5633 *p* = 0.018), and interstitial congestion (for Poractant alfa *p* = 0.040, for CHF5633 *p* = 0.019). Only CHF5633 surfactant significantly affected the presence of hyaline membranes (*p* = 0.029) compared to untreated animals (Fig. 8e–g). Overall, the sum of all observed histological parameters expressed as a total lung injury score was significantly reduced by both surfactant preparations; for Poractant alfa (*p* = 0.009, CI: 1.64, 6.36), and CHF5633 surfactant (*p* = 0.005, CI: 1.64, 6.36) compared to Control group (Fig. 8d). There were no differences in semiquantitative histopathological features between the surfactant preparations.

## Discussion

Acute Respiratory Distress Syndrome (ARDS) presents as a clinical syndrome marked by widespread damage to the alveoli, lung edema, and hypoxemic respiratory failure resulting from either septic or sterile origins. This condition commonly occurs in critically ill patients and is associated with a significant mortality rate (of 30–50%), as highlighted by the recent COVID-19 pandemic, and the substantial number of cases has posed significant challenges for healthcare organizations globally [48]. The pathophysiology of ARDS involves the activation and dysregulation of many interconnected pathways in response to injury, including inflammation and coagulation both in the lung and systemically [8]. Impairment of pulmonary epithelium and endothelium causing damage to the alveolar-capillary barrier is a key factor in ARDS and results in ventilation-perfusion mismatch and subsequent hypoxia [49]. Persistent inflammation, the activation of sPLA, and the leakage of plasma proteins into the alveolar space collectively lead to the inactivation of pulmonary surfactant, resulting in alteration of the surface tension, disturbed gas exchange and the loss of lung function [50, 51]. The objective of treating ARDS is to minimize iatrogenic injury and address the underlying cause. In order to improve oxygenation, invasive mechanical ventilation is the mainstay for most ARDS patients [52, 53] and exogenous surfactant replacement therapy offers another therapeutic option. However, the use of exogenous surfactant in patients with ARDS is still controversial, as studies have produced diverse results. The use of both natural and synthetic surfactant preparations in randomized clinical trials generally resulted in improved oxygenation indices. However, these trials have not demonstrated clear survival benefits [33, 54–56]. Potential explanations for the lack of success in a treatment that holds promise may include variations in surfactant composition, methods of drug delivery, and the diversity in surfactant biology within the target population [57]. In addition, the response to exogenous surfactant in preclinical studies with direct lung injury is promising [44, 58–61].

In this study, we used an in vivo model of severe ARDS to find differences in efficacy between surfactant preparations of different origins on lung function and inflammation that we could not demonstrate in our previous study using a mild ARDS model [43], and an in vitro model of surfactant function to investigate the biophysical activity of exogenous surfactants using a pulsating bubble surfactometer. Impairment of the biophysical activity of surfactants was assessed as minimal surface tension (ST) in the pulsating bubble surfactometer. After 5 min of pulsation, the minimal ST was near 5 mN/m even when HCl (pH 1.25) was added (Fig. 2c). One of the key events in ARDS is an increased alveolar plasma protein load due to increased endothelial and epithelial permeability [18]. Importantly, CHF5633 was significantly inactivated with Alb and Fbg (Fig. 2c). The minimal ST of the samples were high, the border that determines a good biophysical activity of surfactant (over 5 mN/m). Surfactant materials isolated from rabbit lavage fluids were evaluated. The ST values in all groups were below 5 mN/m, including untreated animals (Fig. 2a). We assume that instilation of HCl did not cause a homogeneous distribution and lung damage was not totally diffused. This means that the lungs of injured animals also contained undamaged natural surfactant. In addition, natural surfactant containing all phospholipids and surfactant proteins makes it even more resistant to loss of activity [62].

In an attempt to replicate a clinical scenario capturing the multifactorial etiology of ARDS, two noxious factors causing lung injury were included. Adult rabbits were subjected to a combination of intratracheal instillation of HCl and high-volume ventilation to induce ARDS-like injury. The rationale behind the choice of injurious insults is supported by the fact that (1) aspiration of gastric content is a significant factor leading to ARDS [63], (2) the biophysical forces associated with mechanical ventilation might contribute to VILI [64]. Both injuries lead to loss of microvascular integrity, extravasation of edema fluid and proteins into the alveoli, where they could directly interfere with alveolar surfactant function and affect respiration, as described previously [44]. After induction of lung injury, the lung function parameters of the Control group, such as *P*/*F* and oxygenation indexes, alveolar-arterial gradient, compliance, and oxygen saturation, rapidly deteriorated to levels indicative of intubated severe ARDS (*P*/*F* ≤ 100 mmHg, or ≤13.3 kPa by the new global definition of ARDS [45]) and remained low until the end of experiment (Figs. 3 and 4), consistent with results from previous studies [65–68]. This model should particularly be relevant for the study of pulmonary surfactant inactivation and ARDS that develops secondarily. The combination of damage to the alveolar epithelium leading to altered synthesis, secretion, and breakdown of surfactant with increased functional inhibition creates space for testing exogenous surfactants that are resistant to secondary inactivation [32]. Treatment with both surfactant preparations, CHF5633 or Poractant alfa rapidly improved lung function parameters to a similar extent. These results are similar to previous studies of surfactant therapy in ARDS models [43, 44, 59, 60]. A rapid improvement in *P*/*F*, OI, AaG compared to controls was observed during the first 15 and 30 min after administration with a subsequent reduction in the activity of exogenous surfactants on lung function. This may be due to the inactivation of the therapeutic surfactant in the compromised alveolar space filled with edema fluid, plasma proteins, and activated polymorphonuclear leukocytes. Poractant alfa improved respiratory parameters *P*/*F*, AaG, *C*_stat_, and *P*_aw_ throughout the observation period with a significant effect compared to the control animals.

ARDS is characterized by significant inflammatory cell infiltration and alveolar epithelial and endothelial injury. Lung infection and aspiration of gastric content can directly damage alveolar type-II epithelial cells and impair surfactant metabolism [32]. There is a massive influx of leukocytes especially neutrophils from the circulation into the interstitium and alveolar spaces [69]. In accordance, the histological lung sections revealed an acute cellular response in the alveoli, characterized by a predominance of neutrophils and plasma cells, along with activated pneumocytes four hours after ARDS induction. Activation of these cells is associated with production of pro-inflammatory cytokines such as IL-1β, TNFα, IL-6 and IL-8 [70–72]. Furthermore, activated neutrophils and linked oxidative bursts can cause oxidative damage of proteins and lipids [49, 73]. Since pulmonary surfactant has anti-inflammatory properties, it is desirable to reduce inflammation concurrently with surfactant replacement therapy in ARDS. In this study, both Poractant alfa and CHF5633 surfactant preparations significantly decreased the number of neutrophils in BALF (Fig. 5), and reduced the level of inflammatory and oxidative markers (Fig. 6). Observing the rise in the total white blood cell count in BALF after both surfactant preparations is intriguing. The large and fast changes in WBC count after injury and/or therapy may reflect an early release of leukocytes from the bone marrow and their subsequent trapping in the vessels of the lung. The potent anti-inflammatory and antioxidative properties of exogenous surfactants have been discussed previously [58, 60, 74, 75]. In addition, both surfactant preparations decreased the levels of degrading enzymes causing surfactant dysfunction and endothelial damage (Fig. 6h, i). In particular, reduced sPLA activity after surfactant administration could be informative as it links inflammation and surfactant dysfunction and correlates with clinical outcomes in patients with ARDS [76, 77].

Inflammatory mediators and bioactive substances, including reactive oxygen and nitrogen species, cause damage to endothelial and epithelial cells. Additionally, high lung volumes during model setting result in alveolar rupture, air leakage, and localized lung overdistension [6]. This damage leads to an increased permeability across the alveolar-capillary membrane [78, 79]. Lung wet/dry (*W*/*D*) weight ratio has been shown to reflect the integrity of the alveolar-capillary barrier and the degree of pulmonary edema. Increased lung edema formation and protein content in BALF were observed in control animals. Similarly, massive hyaline membranes, hemorrhage, protein debris, and perivascular edema formation were observed upon histological examination in untreated animals. Both surfactant preparations significantly reduced these changes in lung architecture and resulted in decreased lung edema compared to the controls (Figs. 7 and 8). It could be associated with the reduction of alveolar surface tension, thereby decreasing the tendency of alveoli to collapse and preventing subsequent fluid transudation [80].

The exploration of surfactant replacement as a potential therapeutic approach for ARDS has spanned several decades. Despite the uncertainty surrounding the precise cause of surfactant abnormalities in ARDS and how dysregulated surfactant directly translates to adverse clinical outcomes, it is probable that both surfactant deficiency resulting from direct alveolar epithelial injury and secondary abnormalities involving surfactant functional inhibition due to endothelial leakage play roles in the pathogenesis of ARDS [32]. Serum proteins e.g. albumin may compete with surfactant molecules and reduce surfactant adsorption. Inflammation and the associated oxidation and sPLA can facilitate the hydrolysis of surfactant phospholipids and subsequently alter the fluidity and structure of the surfactant, which significantly affects its biophysical function [81, 82]. Consequently, the exogenous surfactant may restore pulmonary surfactant homeostasis and alveolar epithelial lining integrity. While surfactant replacement therapy is the standard of care in premature neonates with primary surfactant deficiency, studies of adult patients with ARDS so far have demonstrated no survival benefits [32]. Due to the diverse pathophysiology of ARDS, exogenous surfactants used for treatment are likely required to withstand inactivation by the various compounds present in the alveoli to be effective. The most vulnerable to inactivation are surfactant proteins (SP), mainly hydrophobic SP-B and SP-C which substantially enhance surfactant function [83]. Use of synthetic surfactant may provide an alternative to animal‐derived products because natural surfactant is very expensive and large quantities would be required to counteract the effect of surfactant inhibition in adults. Furthermore, current evidence showed the benefits of surfactant use in ARDS if administered a highly functional exogenous surfactant preparation in early respiratory failure [84, 85].

This study has several limitations. First, we used in vivo model of injury from sterile cause e.g. HCl and thus our findings may not be generalizable to clinical settings of gastric juice aspiration cases. Clinical aspiration represents more complex contents e.g. gastric particulate debris, food particles, bacterial products, and cytokine suspensions [70]. Second, we used HCl with a pH 1.25, which is lower than gastric juice pH of ICU patients that ranges from 3.0 to 4.0 [86]. However, pH 4.0 is associated with lung injury and, together with high-volume ventilation mimicking VILI, could represent a valid model of severe ARDS. Third, we altered healthy lung with intact endogenous surfactant and analyzed the effect of surfactant preparation for 4 h, thus the further progress of therapy and injury is unclear. Main focus of the study was on the early and acute phase of ARDS. Fourth, it is obvious to point out several interspecies differences e.g. immune response, differences in the respiratory system structures of animal models and humans [87].

In conclusion, the aim of this study was to compare two surfactant preparations in terms of biophysical function in vitro and biological effects in an animal model of ARDS, focusing on lung function parameters, inflammation, and lung architecture. Using a two-hit model of severe ARDS, more closely reproducing human ARDS, we attempted to distinguish the therapeutic effect of Poractant alfa and CHF5633, exogenous surfactants of different origin. We have shown that administration of the synthetic surfactant CHF5633 in this model leads to a transient improvement in lung function, a reduction in lung inflammation and edema formation, similar to the effects of natural surfactant Poractant alfa. The pathogenesis of the early exudative phase of ARDS includes not only surfactant dysfunction, but also prominent aspects of inflammation, and alveolar-capillary membrane injury. Currently, randomized controlled trials do not endorse the regular application of surfactant. However, increased stability against inactivation, simpler and homogeneous production are the trump cards provided by synthetic surfactants. It seems that synthetic surfactants containing two hydrophobic surfactant peptides or a combination thereof and a more complex phospholipid composition will be able to replace natural surfactants, but further research in this field is necessary.

## Data Availability

The data are available on request from corresponding author.
